# Current grading of prostate cancer

**DOI:** 10.1007/s00292-025-01450-w

**Published:** 2025-07-30

**Authors:** Yosamin Gonzalez Belisario, Geert J. L. H. van Leenders

**Affiliations:** Department of Pathology, room Be-222, Erasmus MC Cancer Institute, University Medical Centre, P.O. Box 2040, 3000 CA Rotterdam, The Netherlands

**Keywords:** Growth pattern, Cribriform pattern, Intraductal carcinoma, Classification system, Gleason score, Wachstumsmuster, Kribriformes Muster, Intraduktales Karzinom, Klassifizierungssystem, Gleason-Score

## Abstract

The Gleason grading system is the cornerstone of risk stratification and clinical decision-making in prostate cancer patients. In the last decade, new insights on quantitative grading and the importance of particular growth patterns have further optimized pathological tumor grading. This review provides an update on the latest grading recommendations and discusses contemporary research directions.

## Abstract (de)

Das Gleason-Graduierungssystem bildet die Basis der Risikostratifizierung und der, klinischen Entscheidungsfindung bei Prostatakrebspatienten. In den letzten zehn, Jahren haben neue Erkenntnisse über die quantitative Einstufung und die Bedeutung, bestimmter Wachstumsmuster die histopathologische Charakterisierung von Tumoren, weiter optimiert. In dieser Übersichtsarbeit werden die neuesten Empfehlungen für die, Graduierung vorgestellt und aktuelle Forschungsrichtungen erörtert.

Prostate cancer (PCa) is the most frequently diagnosed malignancy and is the fifth leading cause of cancer-related mortality among European men. Since more than half a century, the Gleason grading system has been essential for risk stratification and clinical decision-making in PCa patients. Several modifications of the Gleason score (GS) have been implemented over the years. This review provides an overview of the most recent grading recommendations and highlights future research directions.

## Gleason score classification and modifications

In 1966, the Veteran’s Administration Cooperative Urological Research Group (VACURG) designed studies to compare various treatment modalities for PCa, including the assessment of a wide range of potentially prognostic parameters. One of the key components was pathological tumor grading, which was meticulously performed by Dr. Gleason, the trial’s designated pathologist [1]. It was recognized that tumor growth patterns were more important for prognosis than mitotic counts or cytological features. Due to their comparable prognostic performance, specific growth patterns were merged into five Gleason patterns (GP) ranging from well-differentiated densely packed glands to undifferentiated carcinoma with minimal to no glandular formation. Since PCa was frequently composed of more than one GP, Dr. Gleason developed a classification system adding the predominant primary pattern and secondary pattern into a GS ranging from 2 to 10.

The diagnostic assessment in Gleason’s days was quite different from current practice in the 21st century. Prostate cancer was frequently diagnosed either at a late stage due to clinical symptoms or detected incidentally during transurethral resection of the prostate (TURP) performed for lower urinary tract symptoms. Moreover, serum prostate-specific antigen (PSA) measurement and immunohistochemistry were not part of clinical workup at that time. One can imagine that some well-differentiated GS < 4 tumors diagnosed on TURP in the 1960–80s would now appear to represent adenosis/atypical adenomatous hyperplasia, for which distinction via basal cell immunohistochemistry is mostly mandatory.

In 2005, the International Society of Urological Pathology (ISUP) held a consensus meeting which significantly updated Gleason grading recommendations based on the latest scientific evidence and altered clinical practice, thus resulting in a modified GS [2]. For instance, it was consented that GS < 5 should “rarely, if ever” be assigned on needle biopsy. Furthermore, most cribriform patterns were to be classified as GP4, with only a few exceptions of small cribriform glands still being compatible with GP3 [2].

At the subsequent 2014 ISUP meeting, some minor modifications were introduced, most importantly that small cribriform-pattern and glomeruloid glands should now also be labelled GP4 [3]. In addition, a new subclassification proposal of Dr. Epstein’s group was embraced to report ISUP grade groups (GG) in conjunction with the GS: GS ≤ 6 (GG1), GS3 + 4 = 7 (GG2), GS4 + 3 = 7 (GG3), GS8 (GG4), and GS9–10 (GG5) [4]. The clinical benefits of the new GGs were their clear distinction between GS3 + 4 and 4 + 3 and the assignment of GG1 to the lowest-risk tumors instead of GS6.

**Fig. 1:**
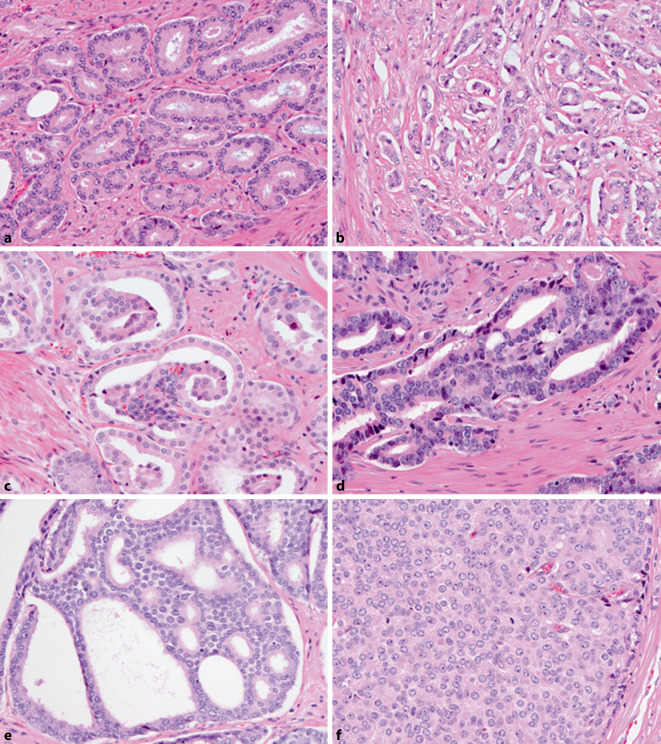
Overview of PCa Gleason patterns (GP). **a** GP3 consisting of well-formed tubules. **b**–**e**: GP4 poorly formed (**b**), glomeruloid (**c**), fused (**d**), and cribriform (**e**). **f** GP5 solid pattern. Hematoxylin and eosin, 200 ×

**Fig. 2:**
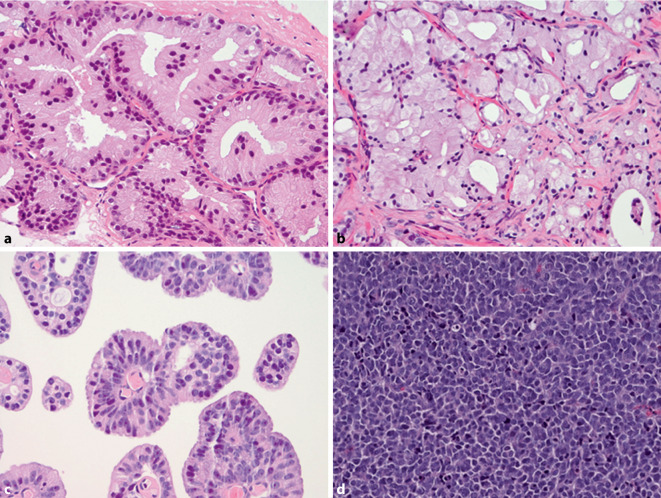
Overview of unusual histological prostate cancer patterns (**a**,**b**) and types (**c**,**d**). **a** Pseudohyperplastic cancer consisting of closely packed atypical glands with subtle papillary infoldings. **b** Foamy gland cancer with abundant foamy cytoplasm and eccentrically placed small nuclei. **c** Ductal adenocarcinoma with papillary structures covered by high-columnar cells with elongated nuclei, graded as GP4. **d** Small cell neuroendocrine carcinoma, which should not be graded. Hematoxylin and eosin, 200 ×

Since the 2014 ISUP consensus meeting, the GGs have encompassed the following growth patterns: GG1 is characterized by the presence of individual, discrete, well-formed tumor glands; GG2 is a mixture of those well-formed glands with a secondary component of poorly formed, fused, glomeruloid, or cribriform GP4 glands, whereas in GG3, GP4 is the predominant pattern; GG4 represents the most heterogeneous grade category, encompassing not only GS4 + 4 = 8, 3 + 5 = 8, and 5 + 3 = 8, but also exhibiting a large variety of relative GP percentages and individual growth patterns; and GG5 includes single cells, cords, nests, solid fields, or the presence of comedonecrosis, which are all labeled GP5, alone or in combination with GP4 (Fig. 1; [3]). Some unusual histological patterns of acinar adenocarcinoma are recognized, including atrophic, foamy gland, pseudohyperplastic, mucinous, and prostatic intraepithelial neoplasia (PIN)-like PCa, and these should be graded based on their underlying architectural pattern, mostly being GP3 (Fig. 2a, b). Apart from these archetypal acinar PCa growth patterns, rare papillary acinar adenocarcinoma and ductal-type PCa should also be labelled GP4 (Fig. 2c; [5]). Small cell neuroendocrine and sarcomatoid carcinoma which predominantly occur at late stage, are high-grade by definition and should not be graded according to the GS (Fig. 2d).

## Quantitative Gleason pattern assessment

Consideration of GP proportions represents the cornerstone of the GS. This is best illustrated in GS7, where the proportions of GP3 and 4 determine whether a tumor is assigned GG2 (3 + 4) or GG3 (4 + 3). However, areas of ambiguity do exist, for instance regarding whether to account for minimal GP volume; co-existence of GP3, 4, and 5; and grading rules for biopsy versus radical prostatectomy (RP) specimens.

The last 2019 ISUP consensus meeting provided the following recommendations on these proportional grading issues [6]:

- In the case that GP3 represents less than 5% of the tumor volume, it is not included in the GS/GG of biopsy and RP specimens.
- In biopsies with a small percentage of higher-grade pattern, this is always included as the secondary grade of the GS, while in RP specimens, a threshold of ≥ 5% should be exceeded.
- If the high-grade pattern is less than 5% in RP, it should be omitted from the GS/GG, but its presence should be reported as a minor or tertiary pattern. For instance, for a single nodule exhibiting 60% pattern 3, 25% pattern 4, and 15% pattern 5, the final GS would be 3 + 5 = 8 in both biopsy and RP specimens [7]. A tumor with 60% pattern 3, 38% pattern 4, and 2% pattern 5 would be graded GS3 + 5 = 8 (GG4) on biopsy, but as 3 + 4 = 7 (GG2) with minor/tertiary component pattern 5 (2%) on RP.

Whereas GP proportions and cut-offs determine the categorization of GS and GG, information on exact GP quantity has added value for risk stratification. In a large study including more than 12,000 RP specimens, Sauter et al. showed that the risk of biochemical recurrence gradually increments with increasing GP4 percentage [8]. Based on this and other studies, the World Health Organization (WHO), ISUP, and the International Collaboration of Cancer Reporting (ICCR) recommend reporting of the percentage of GP4 in GS7 PCa patients [5, 6, 9, 10]. Apart from having prognostic information, the percentage of GP4 in GS3 + 4 = 7 (GG2) is used as an eligibility criterium for some active surveillance protocols [11].

### Research directions

Notwithstanding including the percentage of GP4 in GS7 (GG2-3) pathology reports, overall consideration of the relative GP4 and 5 percentages allows for better clinical risk stratification. To account for continuous relative proportions, the Martini Klinik group in Hamburg has developed a numerical “integrated quantitative Gleason score (IQ-Gleason)” ranging from 0 (100% GP3) to 117.5 (100% GP5) [12]. The discriminative value of the IQ-Gleason score for postoperative biochemical recurrence is better than with current Gleason grading and has been validated independently [13].

Apart from its better performance, continuous grading has another advantage, namely that it is less susceptible to interobserver variability. For instance, consider a PCa biopsy sample with 96% GP3 and 4% poorly formed cords with sparse lumen formation, where one may doubt between assigning GP4 or 5. Assignment of GP4 would result in GS3 + 4 = 7 (GG2) with 4% pattern 4 and imply that one could be eligible for active surveillance. On the other hand, if one would conclude that the respective pattern is better compatible with GP5, this would lead to GS3 + 5 (GG4), which is considered high-risk disease and requires active treatment. Unfortunately, such extreme scenarios are not hypothetical, but rather part of daily grading practice. However, applying a continuous scoring strategy would be more robust, e.g., the IQ-Gleason of the first scenario would be 4 out of 117.5 and of the second consideration 14/117.5, thereby diluting the effects of arbitrary grading variability.

Another recent area of research in grade quantification is whether the absolute GP4 volume is more informative than its relative proportion compared to GP3. For instance, a GS4 + 3 = 7 (GG3) biopsy sample with 2 mm PCa composed of 70% GP4 and 30% pattern 3 has 1.4 mm absolute pattern 4 length. However, a GS3 + 4 = 7 (GG2) biopsy with 12 mm PCa consisting of 80% pattern 3 and 20% pattern 4 has 2.4 mm pattern 4. Although the absolute GP4 volume is higher in the second case, counterintuitively, its assigned GS/GG is lower. Dean et al. compared GP4 quantification methods in GS3 + 4 = 7 (GG2) biopsies with respect to the presence of adverse features at subsequent RP, defined as ≥ GS4 + 3 = 7, ≥ pT3, and/or lymph node metastasis [14]. They found that the overall GP4 percentage, maximum GP4 percentage, and cumulative GP4 length were all significantly associated with adverse pathology, but that the absolute total GP4 length had the best performance and highest net benefit.

In a recent study, Vickers et al. also concluded that quantifying the absolute amount rather than the percentage of GP4 is informative for predicting adverse pathology, and they even found that the GP3 volume did not have a significant impact on outcome [15]. The findings presented above advocate for including GP4 quantification in pathological reports. Although the clinical impact might rely on the absolute GP4 volume rather than its proportion relative to GP3, it is not necessary to report the absolute GP4 length at this moment, because of the scarcity of comparative studies, the absence of standards for uniform GP4 length calculation and reporting, and the unclear consequences for clinical decision-making.

## Invasive cribriform pattern

In has become clear in the past 10 years that among the GP4 growth patterns, cribriform carcinoma is an important and independent adverse pathological parameter regarding clinical outcome. For instance, Kweldam et al. demonstrated that patients with the cribriform pattern in diagnostic biopsies had significantly worse disease-specific survival than those without [16]. The presence of the cribriform pattern has been independently correlated with advanced stage, postoperative biochemical recurrence, radiotherapy failure, lymph node metastasis, and death [17].

Considering its prognostic and clinical importance, a detailed definition of the cribriform pattern has recently been established [18]: the cribriform pattern is characterized by a confluent or contiguous sheet of malignant epithelial cells featuring multiple intercellular glandular lumina that are readily observable at low power (10 × objective magnification) and there should be no intervening stroma or mucin separating individual or fused glandular structures. Consequently, it was consented in the 2019 ISUP meeting and by the WHO in 2022 that the presence of the cribriform pattern should always be reported [5, 6].

### Research directions

Merely its presence and not the extent of the cribriform pattern is associated with an adverse clinical outcome, meaning that even one convincing cribriform gland is a sign of aggressive disease [16, 17]. Whether the size or diameter of a cribriform field has additional prognostic impact remains to be determined. Some studies have found that large cribriform glands are associated with worse outcomes than small fields of cribriform pattern [19, 20].

However, the definition of a large field of cribriform pattern varies among studies, including >12 intercellular luminal spaces, exceeding 0.25 mm, or being twice the size of adjacent benign glands [19–22]. For instance, Greenland et al. showed that biopsy GG2 patients with a large field of cribriform pattern more frequently had adverse pathology at RP than those with small or no cribriform glands [22]. On the other hand, we found that a large field of cribriform pattern at RP was associated with adverse features and postoperative biochemical recurrence, but that small and large fields of cribriform pattern at biopsy had similar independent prognostic value for metastasis-free or specific disease-free survival [20, 23].

Incorporation of the cribriform pattern into novel PCa grading models results in better discriminative value for clinical outcome than current Gleason grading. For instance, we have shown that a simple cGrade model, i.e., decreasing the GG by one, except for current GG1, if no cribriform pattern is present, has better performance for predicting metastasis-free survival than GG [24]. Furthermore, a recent multicenter study showed that including the cribriform pattern improved the performance of the National Comprehensive Cancer Network (NCCN) and Cancer of the Prostate Risk Assessment (CAPRA) risk stratification schemes [25].

## Intraductal carcinoma

The concept of intraductal carcinoma (IDC) was introduced by McNeal et al. in 1986 [26]. These authors defined IDC as a “complete spanning of the ductal or acinar lumen by several trabeculae of malignant epithelial cells, with foci of trabecular fusion.” They found the prognosis of patients with IDC to be equivalent to that of those with GP4. In 2006, Guo and Epstein instituted strict morphologic criteria for the diagnosis of IDC in needle biopsies, i.e., an intraductal proliferation of malignant prostatic epithelial cells with any of the following patterns: solid, dense cribriform, and/or loose cribriform/micropapillary pattern with either marked nuclear atypia or nonfocal comedonecrosis [27]. Numerous studies have demonstrated a strong association between the presence of IDC and high GS, large tumor volume, positive surgical margins, biochemical recurrence, and disease-specific survival [16, 17, 27].

Although invasive cribriform GP4 and IDC are strictly two separate pathological entities, they show significant morphological overlap and often coincide [16]. As demonstrated by several groups, the presence of invasive cribriform carcinoma and IDC are both significantly associated with worse disease-specific survival in uni- and multivariable analyses [16, 17]. Because of their morphological overlap and similar association with adverse outcomes, the 2019 ISUP meeting consented that distinction between them is not necessary if adjacent invasive cancer is present. This implies that pathologists may simply report the presence of “invasive cribriform and/or intraductal carcinoma.” Another implication is that IDC, if present adjacent to invasive cancer, can be graded as if it were invasive cancer. For instance, a biopsy with 90% GP3 and 10% IDC can be reported as GS3 + 4 = 7 (GG2) with 10% GP4 and the presence of invasive cribriform and/or intraductal carcinoma. In the rare case of biopsies containing IDC only without invasive cancer, IDC should not be graded but requires a comment that the single finding of pure IDC is mostly a feature of unsampled high-grade disease. Immediate biopsy should be recommended, and some institutes would even start immediate therapy.

## Atypical intraductal proliferation

The most critical differential diagnosis of IDC at biopsy is high-grade PIN (HGPIN). Both entities exhibit atypical cytologic features such as nuclear enlargement, hyperchromasia, and enlarged nucleoli. While solid and dense cribriform patterns in distended pre-existent glands are not architectural features of HGPIN, the diagnostic classification of loose cribriform structures and cribriform pattern in non-distended glands has been uncertain. Such ambiguous lesions intermediate between HGPIN and IDC have been labelled atypical intraductal proliferation (AIP) [28, 29]. Shah et al. found that biopsy AIP was mostly predictive of unsampled IDC and proposed immediate re-biopsy to confirm the presence of intermediate- or high-grade cancer [28]. Since AIP is not a nosological entity but rather represents a label of uncertainty, like atypical small acinar proliferation (ASAP), reporting and classification recommendations of AIP will likely mature in the future.

## Current developments

Based on an analysis of the prognostic significance of various morphological patterns, McKenney et al. [30] developed a simplified grading model that only distinguishes between favorable and unfavorable patterns. Unfavorable patterns included any components of Gleason pattern 5, large cribriform glands, intraductal carcinoma (IDC), highly stromogenic carcinomas, complex intraluminal papillary architecture, and carcinomas with complex anastomosing growth. This signature demonstrates a high prognostic value with respect to biochemical recurrence, metastasis, and overall survival, but it requires further validation. It remains unclear whether these findings, which were derived from radical prostatectomy specimens, can be directly applied to needle biopsies [31]. Finally, the clinical application of artificial intelligence (AI) in diagnostic prostate histopathology holds great promise. The AI-based algorithms for Gleason grading show high concordance with pathologists, can reduce interobserver variability, and assist in the detection of cribriform growth patterns [32–34].

## Practical conclusion

- The Gleason score is key to risk stratification and clinical decision-making in men with prostate cancer.
- Integrating GP4 quantification into pathology reports and risk models optimizes the prediction of outcome and may be relevant for assessment of surveillance eligibility.
- Invasive cribriform pattern and invasive ductal carcinoma are two morphologically overlapping features independently associated with poor disease-specific survival outcomes.
- Although invasive cribriform carcinoma and intraductal carcinoma are strictly speaking separate entities, they show strong morphological overlap and frequently coexist.
- The most important differential diagnosis of intraductal carcinoma is high-grade prostatic intraepithelial neoplasia.
- Artifical intelligence has huge potential for supporting pathologists in future grading.

## Declarations

For this article no studies with human participants or animals were performed by any of the authors. All studies mentioned were in accordance with the ethical standards indicated in each case.

The supplement containing this article is not sponsored by industry.
